# Physical activity and FTO genotype by physical activity interactive influences on obesity

**DOI:** 10.1186/s12863-016-0357-6

**Published:** 2016-02-24

**Authors:** Joon Young Kim, Jacob T. DeMenna, Sobha Puppala, Geetha Chittoor, Jennifer Schneider, Ravindranath Duggirala, Lawrence J. Mandarino, Gabriel Q. Shaibi, Dawn K. Coletta

**Affiliations:** Kinesiology Program, School of Nutrition and Health Promotion, Arizona State University, Phoenix, AZ USA; College of Health Solutions, School for the Science of Health Care Delivery, Arizona State University, 550N. 3rd Street, Phoenix, 85004 AZ USA; Department of Genetics, Texas Biomedical Research Institute, San Antonio, TX USA; Department of Nutrition and UNC Nutrition Research Institute, University of North Carolina at Chapel Hill, Kannapolis, NC USA; South Texas Diabetes and Obesity Institute Edinburg Regional Academic Health Center University of Texas Health Science Center at San Antonio, San Antonio, TX USA; Mayo/ASU Center for Metabolic and Vascular Biology, Phoenix, AZ USA; College of Nursing & Health Innovation, Arizona State University, Phoenix, AZ USA

**Keywords:** Genetic variations, Physical activity, Obesity-related phenotypes, Latinos

## Abstract

**Background:**

Although the effect of the fat mass and obesity-associated (FTO) gene on adiposity is well established, there is a lack of evidence whether physical activity (PA) modifies the effect of FTO variants on obesity in Latino populations. Therefore, the purpose of this study was to examine PA influences and interactive effects between FTO variants and PA on measures of adiposity in Latinos.

**Results:**

After controlling for age and sex, participants who did not engage in regular PA exhibited higher BMI, fat mass, HC, and WC with statistical significance (*P* < 0.001). Although significant associations between the three FTO genotypes and adiposity measures were found, none of the FTO genotype by PA interaction assessments revealed nominally significant associations. However, several of such interactive influences exhibited considerable trend towards association.

**Conclusions:**

These data suggest that adiposity measures are associated with PA and FTO variants in Latinos, but the impact of their interactive influences on these obesity measures appear to be minimal. Future studies with large sample sizes may help to determine whether individuals with specific FTO variants exhibit differential responses to PA interventions.

**Electronic supplementary material:**

The online version of this article (doi:10.1186/s12863-016-0357-6) contains supplementary material, which is available to authorized users.

## Background

There has been a global increase in the prevalence of obesity across all age groups and it has become a serious health problem as it contributes to the increasing burden of obesity-related comorbid health conditions such as type 2 diabetes and cardiovascular disease [1]. It is well established that individual susceptibility to obesity is determined by the interplay between a genetic component and environmental factors [2]. Since the first identification of the fat mass and obesity-associated (FTO) as an obesity-susceptibility gene by Frayling et al. [3], numerous genome-wide association studies (GWAS) have been performed in order to confirm the association between FTO single nucleotide polymorphisms (SNPs) and obesity-related phenotypes in several populations [4, 5]. More recently, we demonstrated significant heritability estimates of obesity-related phenotypes in our Latino population, and confirmed strong associations to obesity-related traits for the FTO SNPs [6].

With consistent replications of the associations between FTO genetic variants and obesity-related phenotypes, it is of growing interest whether these genetic effects on obesity are modified by lifestyle factors such as physical activity (PA). Although some studies have not observed interaction effects between PA and the FTO SNPs on obesity [7, 8], a detailed meta-analysis by Kilpelainen et al. supports the rigid evidence that PA attenuates the effect of the FTO genetic variants on obesity by analyzing data from 218,166 adults from 45 studies [9]. However, to date, there is a lack of evidence in Latinos who are known as a higher risk population for obesity and type 2 diabetes [10]. Although there are a handful of studies with mixed populations, including Latinos, these studies including our own [6], have only examined the association between FTO genetic variants and obesity-related phenotypes such as body mass index (BMI), waist circumference, or body composition [5, 11–13].

Therefore, the purpose of this study is to examine whether regular PA modifies the effect of the variations in the FTO gene on obesity risk, as measured by BMI, fat mass, hip circumference, and waist circumference in a Latino population.

## Results

The descriptive characteristics of the study participants have been reported previously [7]. Briefly, among 667 participants with mean age of 31.7 ± 13.4 (aged 7–85 years old), 80 % of the study population were adults (>18 years old) and 61 % were female. The prevalence of type 2 diabetes in our population was 12.3 and 34 % of the participants were classified with prediabetes (impaired fasting glucose or impaired glucose tolerance). The FTO SNP association analysis data from our previous study [7] demonstrated that the three FTO SNPs (rs3751812, rs8050136, and rs9939609) were in the HWE and in strong LD with r^2^ ranging from 0.87 to 0.98. The minor allele frequencies of the three FTO SNPs ranged from approximately 23 to 26 %. Briefly, as shown previously [7], heritability estimates for BMI, hip circumference, and waist circumference were significantly moderate in magnitude (range 0.34–0.39) and those phenotypes were significantly associated with FTO SNPs (rs3751812, rs8050136, and rs9939609). In addition, in the current study, heritability estimates for fat mass was determined using SOLAR and was also moderate in magnitude and significant (h^2^ = 0.43 ± 0.11, *P* < 0.0001). Minor alleles of the three FTO SNPs (rs3751812 [T], rs8050136 [A], and rs9939609 [A]) were significantly associated with higher levels of fat mass (all *P* < 0.01). There were no significant association between three FTO SNPs and PA [PA-Yes and PA-No] (rs3751812: *P* = 0.645, rs8050136: *P* = 0.541, and rs9939609: *P* = 0.538).

### Effect of PA on obesity-related phenotypes

The descriptive characteristics by PA group are presented in Table 1. The number of participants who reported that they participated in regular PA was 389 (58.9 %), while 271 (41.1 %) reported that they did not participate in regular PA. Participants who participated in regular PA were slightly younger and more likely to be males (Table 1). After adjusting for the covariate effects of age and sex, participants who engaged in the regular PA exhibited significantly lower BMI, fat mass, hip circumference and waist circumferences, all *P* < 0.05 when compared to those who did not engage in the regular PA. Likewise, in all heritability analyses, which accounted for age and sex effects, the covariate screening found that PA is a strong correlate of BMI (*P* = 0.002), fat mass (*P* = 0.00007), hip circumference (*P* = 0.004), and waist circumference (*P* = 0.00006).

**Table 1:** Descriptive characteristics of participants by groups (“yes” responders vs. “no” responders at regular PA questionaire)^a^

	PA-Yes (*n* = 389)	PA-No (*n* = 271)	Total (*n* = 660)	P-value
Age (years)	31.1 ± 13.6	33.1 ± 13.0	31.7 ± 13.4	0.053
Sex (male/female), *n* (%)	186 (48)/203 (52)	71 (26)/186 (74)	260 (39)/407 (61)	<0.001
BMI (kg/m^2^)^b^	27.9 ± 6	30.1 ± 7.3	28.8 ± 6.6	<0.001
Fat mass (kg)^b^	20.4 ± 10.3	25.4 ± 12.1	22.4 ± 11.3	<0.001
Hip circumference (cm)^b^	104.6 ± 12.4	108.5 ± 14.6	106.1 ± 13.5	<0.01
Waist circumference (cm)^b^	93.3 ± 15.2	98.5 ± 16.8	95.3 ± 16.1	<0.001

Data are mean ± SD unless otherwise indicated. A total of 667 participants participated in the study. Of the 667 participants, data for PA questionnaire, body mass index (BMI), fat mass, hip circumference and waist circumference were not available for 7, 3, 8, 7, and 7, respectively

^a^Not accounted for the relatedness of study participants; ^b^Adjusted for age and sex effects

### Interaction effect of the three FTO SNPs and PA on obesity-related phenotypes

**Fig. 1:**
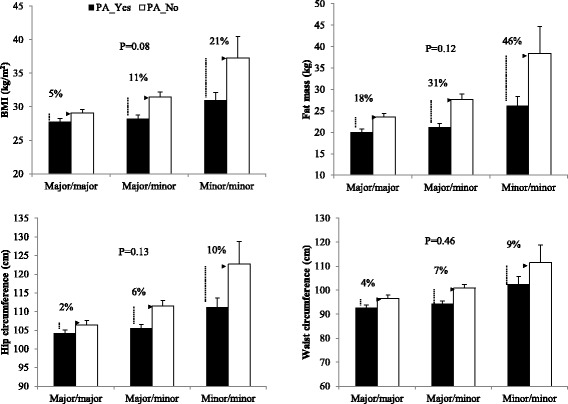
Interaction between PA and FTO SNP rs3751812 on adiposity-related phenotypes. Major allele (G)/minor allele (T); minor allele T considered risk allele

Table 2 describes the interaction effects of the three FTO SNPs and PA on BMI, fat mass, hip circumference, and waist circumference after adjusting for age and sex. None of the interaction terms were found to be significant (*P* < 0.05) as revealed by the genetic analyses. However, a trend can be seen toward significance in the interaction effects between the three FTO SNPs and PA on BMI, fat mass, and hip circumference. For example, regarding the SNP rs3751812, its interactive influences with PA on BMI are suggestive (*P* = 0.08). As shown in Table 2 and Fig. 1, a trend can be observed wherein the carriers of FTO minor (risk) alleles at the three examined loci who are engaged in regular PA are at reduced obesity risk compared to those rare variants carriers who are associated with decreased levels of PA. For example, regarding the marker rs3751812, after taking the SNP and interaction influences into account, the carriers of risk allele “T” engaged in regular PA exhibited reduced obesity risk as shown by the effects in units of BMI (SE) by risk allele carrier status compared to those not engaged in regular PA: Heterozygous carriers: PA-Yes = 0.855 ± 0.254 versus PA-No = 2.729 ± 0.703 and Homozygous carriers: PA-Yes = 1.791 ± 0.507 versus PA-No = 5.457 ± 1.406.

**Table 2:** Interaction between PA and FTO SNPs on adiposity-related phenotype

SNP rs no.	Phenotype	Major/major		Major/minor		Minor/minor		P^a^
		PA_Yes	PA_No	PA_Yes	PA_No	PA_Yes	PA_No	
rs3751812 (G/**T**)	BMI	27.8 ± 6.1	28.9 ± 6.4	28.2 ± 5.7	31.2 ± 7.1	30.9 ± 4.8	37.3 ± 8.3	0.08
	Fat mass	20.2 ± 10.9	24.2 ± 11.6	21.1 ± 9.7	27.5 ± 12.8	26.3 ± 8.5	38.4 ± 16.5	0.12
	HC	104.2 ± 12.7	106.5 ± 13.3	105.5 ± 11.5	110.9 ± 13.7	111.2 ± 9.8	122.7 ± 16.1	0.13
	WC	92.7 ± 15.3	96.5 ± 16.3	94.2 ± 14.7	100.4 ± 16.1	102.5 ± 13.3	111.5 ± 19.4	0.46
rs8050136 (C/**A**)	BMI	28.0 ± 6.1	28.9 ± 6.5	27.9 ± 5.7	30.9 ± 7.0	30.7 ± 4.9	34.7 ± 9.0	0.14
	Fat mass	20.5 ± 11.0	24.4 ± 11.8	20.6 ± 9.7	27.1 ± 12.6	25.4 ± 8.6	33.2 ± 17.4	0.10
	HC	104.6 ± 12.5	106.6 ± 13.4	104.8 ± 12.0	110.4 ± 13.7	109.9 ± 10.0	118.3 ± 16.8	0.14
	WC	92.9 ± 15.2	96.8 ± 16.6	93.8 ± 14.9	100.0 ± 15.9	100.7 ± 14.3	105.8 ± 20.8	0.61
rs9939609 (T/**A**)	BMI	27.9 ± 6.2	28.8 ± 6.4	28.0 ± 5.7	30.9 ± 7.0	30.6 ± 4.8	34.7 ± 9.0	0.60
	Fat mass	20.4 ± 11.1	24.3 ± 11.8	20.8 ± 9.6	27.1 ± 11.8	25.3 ± 8.4	33.2 ± 17.4	0.17
	HC	104.4 ± 12.8	106.4 ± 13.2	105.0 ± 11.7	110.3 ± 13.6	109.9 ± 9.7	118.3 ± 16.8	0.21
	WC	92.7 ± 15.5	96.6 ± 16.6	94.1 ± 14.6	100.0 ± 15.8	100.2 ± 14.1	105.8 ± 20.8	0.78

Data are mean ± SD unless otherwise indicated. SNP rs no (Major allele/Minor allele) and bold allele indicates risk allele; BMI, body mass index; HC, hip circumference; WC, waist circumference. rs3751812 (major allele [G]; minor allele [T]); rs8050136 (major allele [C]; minor allele [A]); rs9939609 (major allele [T]; minor allele [A])

^a^P-values from the genetic analyses regarding the significance of the intercation terms

When the three genotype categories of a given SNP were dichotomized as risk allele carriers (at least more than one risk allele) vs. no carriers; the carriers of the rare allele who participated in regular PA exhibited more clearer pattern of low obesity risk compared to those carriers that did not engage with regular PA (data not shown). As reported in Fig. 1, individuals with minor/minor alleles (considered as carrying two risk alleles) who were engaged in regular PA exhibited the largest reduction at BMI, fat mass, and hip circumference compared to the counterparts (21, 46, and 10 %, respectively). Thus, the homozygous carriers of the minor alleles with no PA appear to be at greater risk for obesity compared to the others.

## Discussion

In the present study, we demonstrated that the obesity measures BMI, fat mass, hip circumference, and waist circumference are correlated with PA as well as with three FTO variants, and that there was some trend toward significance in the interaction effects between PA and FTO genetic variants on these four obesity-related phenotypes in a Latino population. Participants who did not engage in regular physical activity and were carriers of risk allele (minor allele) in the FTO gene exhibited higher BMI, fat mass, and hip circumference, although such relationships lacked statistical significance. In addition to confirming previous association studies in FTO related obesity risk; our data further suggest that PA may modify the genetic effect of FTO on the obesity-related risk in Latino children and adults, given that the examined FTO variants are not associated with PA in our sample.

FTO is the fat mass and obesity-associated gene but, in spite of its name, the exact physiological function of FTO is not well known. The human FTO gene is a 9-exon gene (covering more than 400,000 base pairs) located on human chromosome 16 and encodes a 2-oxoglutarate-dependent nucleic acid demethylase [14]. It has been widely demonstrated that the FTO gene may play an important role in energy homeostasis by regulating either energy expenditure or energy intake in humans [15, 16]. In addition, FTO has been described as a regulator of adipose tissue metabolism (i.e. lipolysis), as it contributes to the regulation of fat mass [17]. Further, a recent review not only supports the physiological role of FTO, but also introduces the novel biological function of FTO in epigenetic regulation [18]. Continuous efforts have been conducted to further clarify the functional role of FTO and it seems that previous work may reasonably support the mechanism underlying the link between FTO genetic variants and obesity.

The association between FTO genetic variants and obesity has been well replicated and established across ethnicities, but the main focus has been on those of European ancestry [3, 4]. Although limited data have been reported in Latino populations, several studies ( GWAS and replication studies) with mixed populations including Latinos have also exhibited the association between FTO genetic variants and obesity measures [5, 11–13, 19]. Scuteri et al. [5] first found the SNP rs9930506 in relation to BMI, hip circumference, and weight in 839 Hispanic American in the GenNet study. Other groups have attempted not only to replicate previous findings, but also to find more associations of FTO SNPs with other adiposity-associated variables such as computed tomography-derived measures of adiposity [11], adipose tissue distribution [13], and measures of glucose homeostasis [19] in multi-ethnic groups including Hispanic populations. Recently, we demonstrated significant heritability estimates of obesity-related phenotypes in our Latino population, and confirmed strong associations to obesity-related traits for the FTO SNPs [6].

As previously mentioned, lifestyle factors such as PA and diet contribute to the obesity epidemic and may interact with genetic effects to modify the risk for obesity [20]. A growing number of studies have recently examined whether there are interaction effects between those lifestyle factors and FTO genetic variants on obesity risk. Interestingly, in terms of food intake and weight control, many studies have been in agreement with not only the association between FTO genetic variants and food intake [21] or increased appetite [22], but also the interaction effects of diet components with FTO SNPs on obesity risk [23]. In addition, a growing number of studies have reported that physical inactivity confers an increased risk of FTO genetic predisposition to obesity while physically active participants who have risk allele(s) of FTO SNPs exhibited lower risk for obesity [9, 24]. These data suggest that PA may be a moderator of the deleterious (i.e. risk-increasing) effects of FTO genetic variants on the risk for obesity. Our data suggest that regular PA may modify the effect of FTO genetic contribution to the obesity risk including BMI, fat mass, and hip circumference. It is possible that changes in waist and hip circumferences in response to regular PA or exercise may differ by sex [25]. Since the proportion of females in this study was 61 %, we did somewhat expect that the interaction effects of PA may differ between hip circumference and waist circumference.

To our knowledge, the majority of interaction studies have focused on European populations [9, 26] and a lack of data exist in the Latino population, even though it is well established that Latinos are at increased risk for obesity and type 2 diabetes [10]. Therefore, our data are relevant as we exclusively focused on Latino children and adults. In the current study, it is important to note that participants were asked whether they engage in regular PA. We did not measure PA levels nor did we define active or inactive individuals based upon some established reference such as the *2008 Physical Activity Guidelines for Americans*, a scientific evidence-based update on the health benefits of a physically active lifestyle, released by the U.S. Department of Health and Human Services [27]. Our interpretation of moderating regular PA effect on the association between FTO genetic variants and obesity may provide a more realistic message to minority populations, suggesting that regular or habitual PA, of any amount, may be more effective at attenuating the FTO genetic effects on obesity than recommending a specific amount of PA that may be tied to general health [28]. Therefore, from a clinical or public health perspective, our data may help to emphasize the importance of daily healthy lifestyle or behaviors for reducing the genetic predisposition for obesity risk.

Despite these strengths, we do acknowledge potential limitations in our data which should be considered for proper interpretation of our findings. Two notable limitations are the small sample size used to assess interaction influences in this study compared to those used in the current large-scale association studies [29], and the use of the questionnaire-based PA assessment which can be affected by study participant recall bias [30]. It should be noted that the PA questionnaire used in the present study does not allow quantify how much PA is needed to reduce the effect of FTO on obesity-associated phenotypes. Given the issue of small sample size and low power, further studies of a larger cohort are warranted to detect lifestyle effects including PA as a moderator on the genetic effects of obesity. Also, we do not know whether the interaction effects are over- or under-estimated because of the accuracy of PA measurement [31]. Participants’ PA levels in the current study were simply measured by participant recall, which was not objectively quantified. Since our main purpose of the AIR registry is to develop a biorepository to examine cardiometabolic disease risk in the Latino population [32], lack of detailed information exists in the screening of lifestyle or environmental factors such as PA, diet/nutrition, and socioeconomic status. However, our data provide evidence for the utility of a simple PA screener in comparing obesity risk factors between PA groups, suggesting that current PA screeners can be used to explore the interaction effects of genetic variants on the obesity measures.

## Conclusions

Our study highlights the possibility that genetic susceptibility to obesity may be modified by engaging in regular physical activity (regardless of whether an individual meets current PA guidelines). From a public health perspective, our findings are highly relevant with respect to our focus on a Latino population with disproportionately increased risk for obesity and type 2 diabetes [33–36]. It is likely that this group is genetically predisposed to obesity and obesity-related comorbid conditions. Encouraging any physical activity in genetically susceptible individuals in order to mitigate the deleterious effects of obesity on health may be a more effective approach. Future studies are warranted to examine whether there are age- or sex-specific differences in the extent of interaction effects or whether individuals with specific FTO variants exhibit differential response to PA interventions.

## Methods

### Participants

Data from 667 Latino children and adults (aged 7–85 years old) from the Arizona Insulin Resistance (AIR) registry were used in the present analysis. The principal investigator and the ad hoc scientific steering committee of the AIR registry granted access to the specimen and data for this study. A description of the registry, the phenotypic characterization of the AIR participants and the procedures for gaining access to the data have been discussed in detail elsewhere [32]. Briefly, of the 667 participants enrolled in the study, 365 were distributed across 92 families from the AIR registry. The 365 participants from 92 families generated 723 relative pairs that were distributed across fourteen relative-pair categories [6]. The remaining 302 participants were found to be represented by single individuals. The institutional review board of Arizona State University approved all procedures, and all subjects gave informed written consent before any research procedures. Once youth (ages 7–18 year old) were in agreement for the participation, written consents (child assent and parental consent) were obtained.

### Measurements, physical activity questionnaire, and SNP genotyping data

Participants arrived at the Arizona State University Clinical Research Unit after an overnight fast followed by screening of their medical history. Additionally, participants were asked whether they are engaged in regular physical activity (i.e. participants answered either yes or no). A complete intake questionnaire used in the AIR registry is shown in Additional file 1. Six-hundred and sixty participants had complete PA data. Anthropometric measurements included height, weight, BMI, hip circumference, and waist circumference. Bioelectrical impedance analysis was performed to estimate body composition (fat mass). All procedures were approved by the institutional review board of Arizona State University and all participants gave informed written consent before their participation.

Participants were divided into two PA groups (PA-Yes; participants who engaged in the regular PA vs. PA-No; those who did not engaged in regular PA) for analysis of the main effect of PA on obesity risk as well as the interaction effect of PA with FTO SNPs on obesity risk. To normalize the distributions for genetic analyses, BMI, fat mass, waist and hip circumferences were transformed using inverse normalization. Untransformed data are presented for ease of interpretation.

The SNP genotyping association analysis for the FTO variants (rs3751812, rs8050136, and rs9939609) with adiposity measures (excluding fat mass) have been previously published [6]. The FTO SNP data for all participants was used in the present study to determine the interaction effect with PA on measures of adiposity.

### Statistical analysis

All statistical genetic analyses were performed using the variance components (VC) approach as implemented in the software package Sequential Oligogenic Linkage Analysis Routines (SOLAR) [solar.txbiomedgenetics.org] [37]. The heritability of a given phenotype is determined using the VC technique (SOLAR), and a likelihood ratio test is used to test whether the heritability of a given phenotype is significant (*P* < 0.05). Covariates age and sex are simultaneously controlled for in all analyses. In addition, all models included PA as a covariate, and a likelihood ratio test is used to assess its significance (*P* < 0.05).

The associations between the FTO genotypes and adiposity related quantitative traits are examined using the measured genotype approach (variance components [VC] MGA) within the analytical framework, which allows us to account for the non-independence among family members [37, 38]. Our previous association analyses for the FTO variants has been described in detail elsewhere [6]. Briefly, in the VC approach, variance components are modeled as random effects, whereas the effects of measured covariates such as age and sex are modeled as fixed effects on the trait mean. The variant genotypes are incorporated in the mean effects model as a measured covariate assuming additivity of allelic effects [38]. Maximum likelihood techniques are used to estimate the variance components, the association parameters, and the other covariate effects. The hypothesis of no association is tested by comparing the likelihood of a model, in which the effect of the measured genotype is estimated with a model, where the effect of the measured genotype is fixed at zero. We have extended this approach to assess genotype x PA interaction influence on a given obesity measure by allowing SNP genotype, PA, and SNP genotype x PA interaction as fixed effects. All analyses included age and sex terms as additional covariates. The hypothesis of no interaction between SNP genotype and PA on a given obesity measure is tested by comparing the likelihood of a model, in which the effect of the interaction term is estimated with a model, where the effect of the interaction term is fixed at zero. For these analyses (i.e., association or interaction), *P* values < 0.05 are considered statistically significant.

SOLAR was also used to calculate the allele frequencies, to test deviations from Hardy-Weinberg Equilibrium (HWE), and to estimate linkage disequilibrium (LD) between SNP pairs using r^2^ values. For the purpose of illustration, at some instances, data are described using the program SPSS without taking the relatedness of study participants into account.

## Additional file

Additional file 1:
**Intake questionnaire**. (DOC 51 kb)

## Abbreviations

AIR: Arizona Insulin Resistance
BMI: body mass index
FTO: fat mass and obesity-associated
GWAS: genome-wide association studies
HWE: Hardy-Weinberg Equilibrium
LD: linkage disequilibrium
MGA: measured genotype approach
PA: physical activity
SNPs: single nucleotide polymorphisms
SOLAR: Sequential Oligogenic Linkage Analysis Routines
VC: variance components
