# Supplementary material for: Physical activity and FTO genotype by physical activity interactive influences on obesity
Source: BMC Genet. 2016 Feb 24;17:47. doi: 10.1186/s12863-016-0357-6 (PMC4765034; doi:10.1186/s12863-016-0357-6)
Supplement: Additional file 1: — Intake questionnaire. (DOC 51 kb) [file 12863_2016_357_MOESM1_ESM.doc]

**INTAKE FORM**

Today’s Date: M / F BILINGUAL: YES/NO

Date of Birth (month/day/year) ____/___/_____ Age: (Must be ≥ 8) Preferred Language: .

Ethnicity:  Mexican  Central American  South American  Other:

What Country (Include Region &/or City) were you born?_______________ .

When did you move to the US?___________ How did you hear about study? .

What motivated you to participate in study?  Concerned about health?  Referred by Friend/family?

 Financial compensation?  Support research for Latinos?

Medical Conditions:

R/T BY .

R/T BY .

R/T BY .

R/T BY .

R/T BY .

NO AT TIME OF VISIT ****

Medical History Yes No

| Pancreatitis |  |  |
| --- | --- | --- |
| Heart disease |  |  |
| Peripheral vascular disease/atherosclerosis |  |  |
| Renal disease |  |  |
| Liver disease |  |  |
| Anemia |  |  |
| Pulmonary disease |  |  |
| Pulmonary embolus |  | CHILD = CH  PARENT = P  SPOUSE=SP  SIBLING=SI  GRANDPARENT= GP  AUNT/UNCLE= T  COUSIN= CO  NEPHEW/NIECE= N |
| Retinopathy/retinal hemorrhage |  |  |
| Neuropathy |  |  |
| Electrolyte abnormalities |  |  |
| Diabetes (if yes: type 1 or type 2) |  |  |
| Blood clotting disorder |  |  |
| Deep venous thrombosis |  |  |
| Cancer (remission > 3 years) |  |  |
| Pacemaker or other internal electrical medical device |  |  |
| Hypertension |  |  |

Medications: NO/ YES

NAME: DOSAGE: FREQUENCY: TAKEN FOR:

NAME: DOSAGE: FREQUENCY: TAKEN FOR:

NAME: DOSAGE: FREQUENCY: TAKEN FOR: NAME: DOSAGE: FREQUENCY: TAKEN FOR:

Are you pregnant? Yes / No N/A

Previous Pregnancies: Gestational Diabetes: Yes / No

Alcohol consumption: yes no If yes, how much:

Smoking / Tobacco products: yes no

Exercise: yes no if yes, Frequency: ____ / Wk Intensity: low hard mod Duration: ______mins;

Family Hx of type 2 diabetes (Circle): Mother Father Sibling Maternal G-Parent Paternal G-Parent
